# Sustainable Laboratory Capacity Building After the 2014 Ebola Outbreak in the Republic of Guinea

**DOI:** 10.3389/fpubh.2021.659504

**Published:** 2021-06-04

**Authors:** Jean Ndjomou, Scott Shearrer, Brendan Karlstrand, Carmen Asbun, Jesse Coble, Jane S. Alam, Mar P. Mar, Lance Presser, Scott Poynter, Julia M. Michelotti, Nadia Wauquier, Casey Ross, Sharon Altmann

**Affiliations:** ^1^MRIGlobal, Gaithersburg, MD, United States; ^2^MRIGlobal, Kansas City, MO, United States

**Keywords:** laboratory diagnostics, capacity building, biorisk management system, quality systems, infectious diseases, Republic of Guinea, laboratory systems strengthening

## Abstract

**Background:** The 2014–2016 West Africa Ebola virus disease outbreak heavily impacted the Republics of Guinea, Sierra Leone, and Liberia. The outbreak uncovered the weaknesses of the public health systems, including inadequately trained and insufficient health personnel as well as limited and poorly equipped health infrastructures. These weaknesses represent significant threats to global health security. In the wake of the outbreak, affected countries made urgent requests for international engagement to help strengthening the public health systems.

**Methods:** This work describes the successful multi-year implementation of a laboratory capacity building program in the Republic of Guinea. The program integrated biorisk and quality management systems training, infectious diseases diagnostic training, facility engineering and maintenance training, and mentorship to strengthen Guinea's bio-surveillance capacity.

**Results:** The major outcome of these efforts was an established and local staff-operated public health laboratory that performs disease surveillance and reporting and diagnostic of priority diseases and pathogens of security concerns.

**Conclusions:** This work has improved the Guinea country's capabilities to address country public health issues and preparedness to respond to future infectious disease threats.

## Introduction

Infectious agents are permanent threats to global health security and stability (1–3). Sub-Saharan Africa is particularly endangered as a result of close interactions between wildlife and human that favor cross-species transmission with the potential to generate highly pathogenic infections. Moreover, the paucity and fragility of their health systems hamper the readiness of sub-Saharan African nations to adequately respond to infectious threats (4). In past years, sub-Saharan Africa registered most of the disease outbreaks globally (5).

The 2014–2016 Ebola virus disease (EVD) outbreak in West Africa highlighted systemic public health weaknesses and the regional health systems were unable to mobilize adequate resources in time to stop early transmission. It also underscored the necessity for timely international intervention and action to stop outbreaks from spreading across borders. International mobilization helped to control the epidemic but the response was slow, unwieldly, and poorly coordinated (4, 6, 7). The Republic of Guinea (RoG) was severely hit, with at least 2,536 reported deaths (8, 9) and associated economic losses evaluated at 600 million US dollars (10).

The World Health Organization (WHO)'s International Health Regulations (IHR 2005) (11) mandate that Member States develop the core capacities needed to detect, investigate, report, and respond to threats that could constitute public health emergencies of international concern (PHEICs) (12). A 2015 report found, however, that many countries including much of sub-Saharan African still lagged behind in full IHR 2005 implementation even 10 years after the original prescribed 5 year implementation period (13). This gap in the implementation of the IHR 2005 core capacities undoubtedly exacerbated the burden of the 2014 West Africa EVD outbreak. The management of the outbreak was complicated by the severe lack of resources including adequate laboratory capacity that could had allowed timely diagnosis of the index case thereby reducing the outbreak burden. Consequently, in the wake of the outbreak, it became clear that building and strengthening the health systems including clinical and public health laboratories in the affected countries was an urgent need (7, 14). In this context, the RoG's Ministry of Health and Hygiene (MoHH) called on international partners for assistance to help fill the gaps and develop a strong national health system. During the 2014 EVD outbreak, the United States government provided the RoG with a containerized molecular diagnostics laboratory, which was permanently installed in Nongo within the Ratoma commune of the capital city of Conakry. The laboratory was fed with international workers during the outbreak and supported the diagnosis of many cases of EVD. At the end of the outbreak, the laboratory was shut down after international workers returned to their primary duty stations. Prior to starting this project, no operational capacity was available at the laboratory. Furthermore, the laboratory networks in the Republic of Guinea was precarious and still under construction. This laboratory facility was intended to serve as the primary molecular diagnostic and research laboratory for the National Public Health Institute (Institut National de Santé Publique, INSP), making it the country's general public health molecular diagnostic laboratory.

Here, we describe the implementation of a capacity-building program that integrated training and mentorship on biorisk management, quality management, molecular and serological diagnostics, and facility engineering. The goal of this multi-year effort was to help the MOHH establish and operate the INSP Nongo laboratory and lay the groundwork for further development and strengthening of the broader laboratory system. The result of the effort is a Guinean-run and maintained facility with a demonstrated capacity for performing disease surveillance, outbreak response, and diagnostics for infectious disease including Guinean's priority diseases and pathogens that represent major threats to global health security.

## Methods

### Curricula Development and Delivery

The training curricula for laboratory biorisk and quality management were adapted from the Global Biorisk Management Curriculum (GBRMC) and the WHO's Laboratory Quality Management System Training Toolkit (15). These materials were developed to align with key international guidelines and standards of practices, including the CEN Workshop Agreement Laboratory Biorisk Management Standard CWA 15793 (16), the WHO Laboratory Biosafety Manual (17), and the ISO 15189 *Medical laboratories—Requirements for quality and competence* (18). The material was presented through a combination of didactic presentations, small group discussions and activities, demonstrations, and drills.

**Figure 1:**
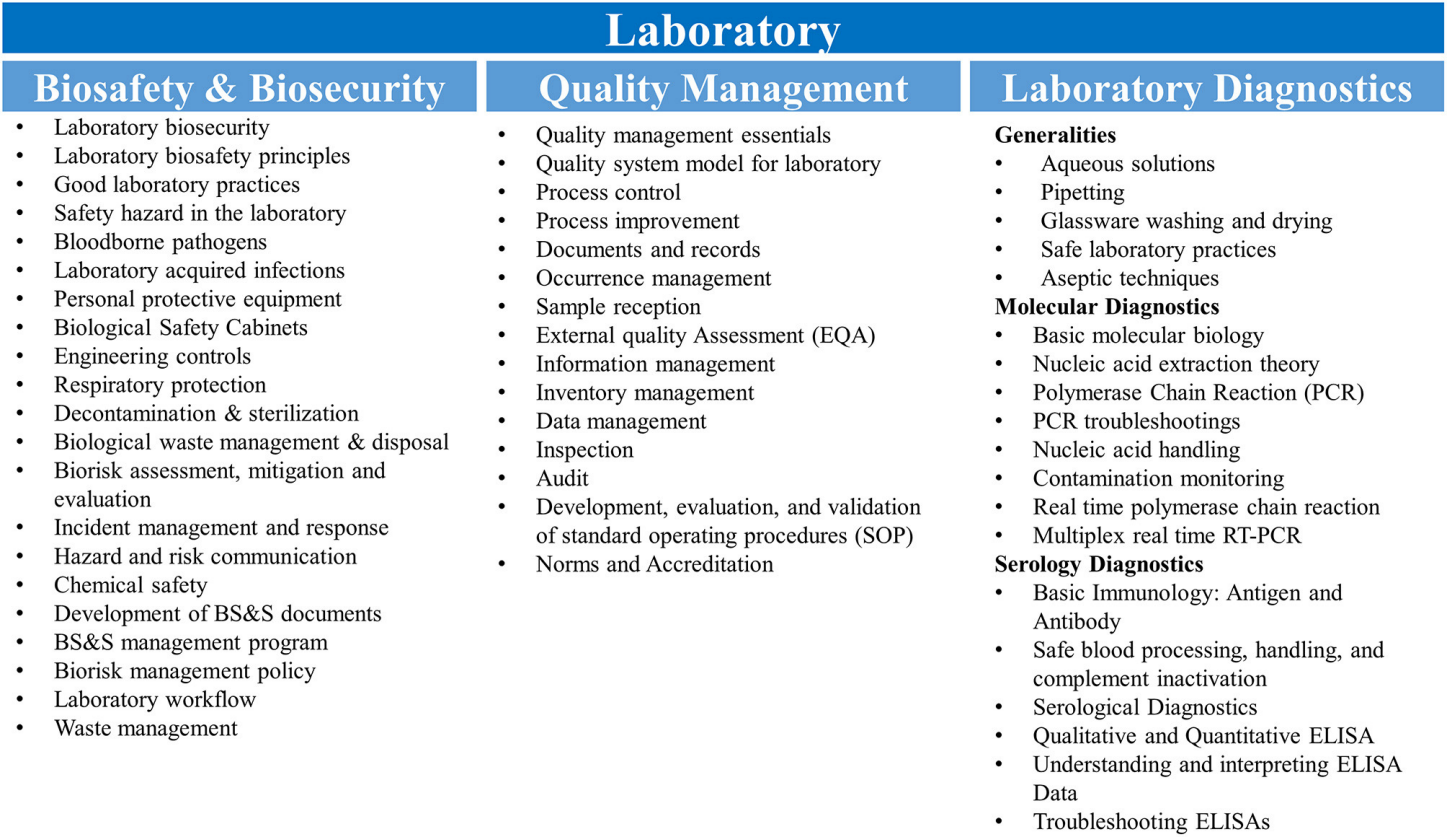
Training curricula. Topics covered for the biosafety & biosecurity, quality management and molecular and serological diagnostics are shown. RT-PCR, reverse transcription polymerase chain reaction; ELISA, enzyme-linked immunosorbent assay.

Molecular diagnostics training simulated the workflow and operations that would be in place during an EVD outbreak. Where possible, a 1:1 trainer:trainee ratio was maintained in order to facilitate individualized instruction and minimize crowding within laboratory spaces. A review of fundamental laboratory skills, molecular biology, and immunology was also included in the staff-level presentations to address knowledge gaps identified during initial engagements with the Guinean laboratorians. Laboratory skills were practiced while wearing the personal protective equipment recommended by a risk assessment performed on conducting EVD diagnostics in the facility to accustom the trainees to integrate biosafety practices with their diagnostic activities. Practical skills development was closely monitored by the trainers and strengthened during mentorship. The diagnostic training was delivered through combination of didactic presentations, review of facility-specific standard operating procedures developed and refined during the EVD epidemic, and simulation exercises designed to develop proficiency across all stages of the diagnostic workflow. The major topics covered for laboratory biosafety & biosecurity, quality management, and laboratory diagnostics are presented in Figure 1.

### Training Period and Participant Recruitment

The training events under this project occurred between 2017 and 2019, with an average duration of 6 weeks of engagement per training event. Biorisk and quality management-related events took place offsite at various hotel conference facilities in Conakry while the molecular diagnostics training occurred at the INSP Nongo laboratory facility. The training efforts prioritized engagement with laboratorians identified by the MoHH and the National Public Health Institute (Institut National de Santé Publique, INSP). Additional training slots were made available to enroll laboratorians from other key public health laboratories within Conakry.

One unique element of this program was the assignment of a full time in-country Program Lead to support the operational standup and running of the Nongo laboratory after the EVD outbreak. The Program Lead mentored the INSP Nongo laboratory and operations staff, and coordinated the training event planning and execution under this project. Mentorship for laboratory personnel started in late 2016 and extended through early 2019. The mentorship included laboratory activity coordination and management, further training and strengthening of the laboratory staff on diagnostic workflows and laboratory work best practices. It also included coordination with US CDC and WHO partners to implement additional diagnostic capacities by providing additional training and mentorship that targeted diagnostic workflows using reagents provided by those partners. The country Program Lead also coordinated and managed training activities and in-country interactions and discussions with representatives of MoHH and other partners.

The training and mentorship of two staff involved in the laboratory facility and equipment maintenance ran from 2017 through 2019. This training and mentorship occurred every 3 months and each visit by MRIGlobal engineers at the INSP Nongo laboratory facility had a duration of 45 days. Monitoring and evaluation of the two individuals also occurred during the visit of the INSP Nongo laboratory facility by MRIGlobal engineers. The Ministry of Health & Hygiene arranged for two graduates of the Higher Institute of Technology (Institut Supérieur de Technologie; IST) of Mamou to support the Nongo laboratory's engineering needs. These two individuals were recent graduates and had no prior experience with medical laboratory facility and equipment maintenance. To familiarize them with the laboratory systems and provide equipment-specific training, the two Guinean engineers received on-the-job training (OJT) from mechanical and electrical engineers familiar with the molecular laboratory's design, construction, and maintenance. The training focused on facility use, maintenance, and safety. Maintenance and repair procedures were taught for multiple laboratory systems, including the ventilation, filtration, and air conditioning systems; site generator, switches, and electrical systems; gray and black waste water systems; cold storage equipment; site incinerator; and laboratory equipment. The two Guinean maintenance engineers also received initial training on performing biosafety cabinet certifications.

### Participant Feedback and Training Evaluation

For training evaluation, questionnaires were designed that allowed the trainees to self-report the impact that the training had on their level of knowledge of the topics addressed during the training sessions (knowledge impact) and the extent to which they implemented (or made the training materials available to or discussed the materials with co-workers) what they learned at the training events upon returning to their home institutions (sustainability). Respondents ranked the impact of the trainings on a scale of 1 (minor impact) to 4 (major impact), and answered yes or no to the sustainability assessments. Respondents were also given the opportunity to provide specific examples of how they had implemented or shared the information they received at the training. Additionally, the capacities of training participants to develop laboratory manuals and standard operating procedures were taken into account. For that, country program Lead and trainers monitored the progress made by institute leaderships and key personnel in appointing biosafety and quality officers, developing biosafety and quality manuals, and writing standard operating procedures for each activity being conducted at the laboratory. This monitoring assessment was done at the INSP laboratory and the Central Veterinary Diagnostic Laboratory (CVDL) at the end of the training sessions through audit visits at the laboratories. Indicators of the assessment were formal appointment of Quality and Biosafety Officers, availability of quality manuals and standard operating procedures (SOPs) for tests performed at CVDL and INSP.

## Results

### Participant Recruitment

**Figure 2:**
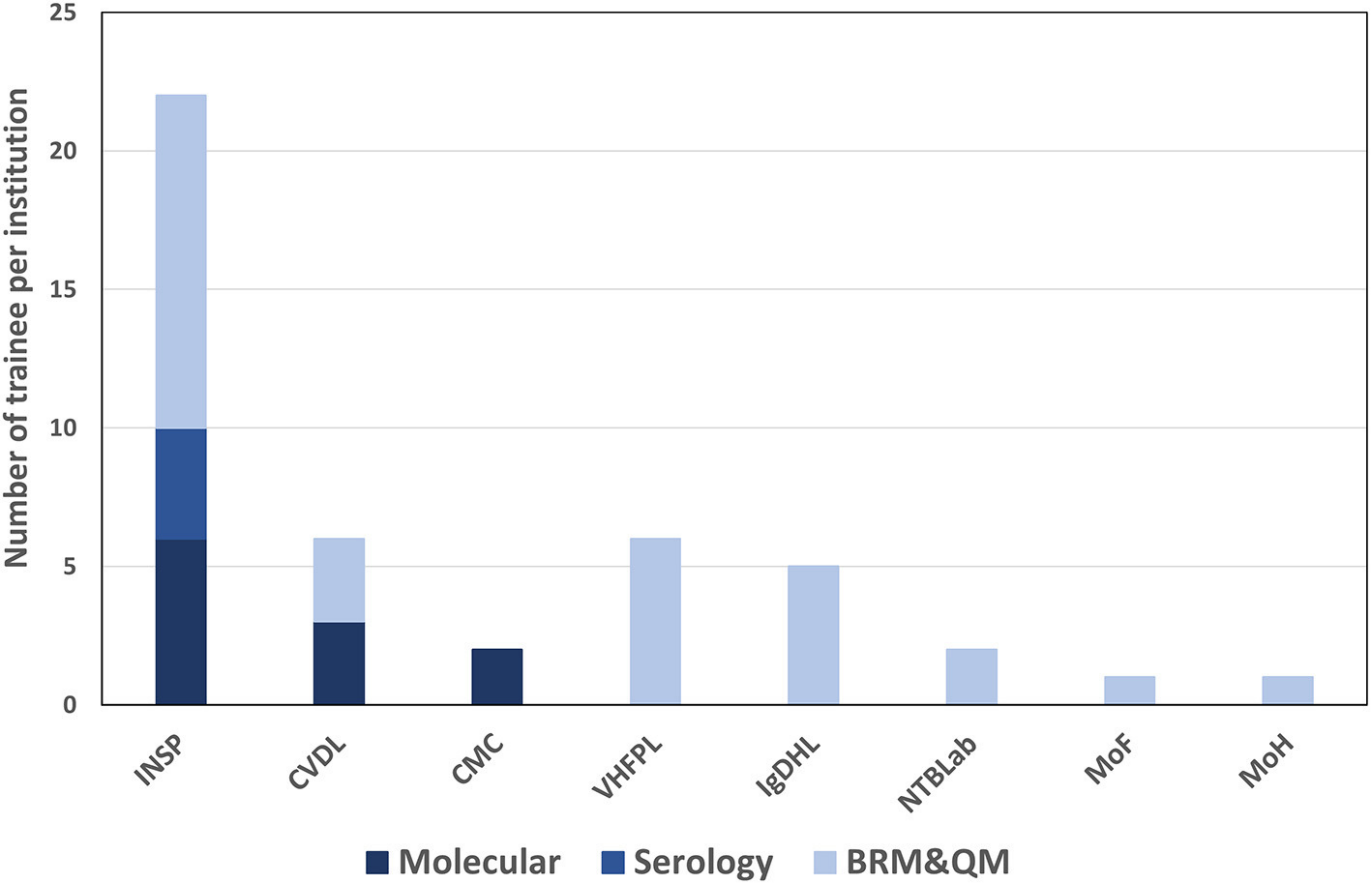
Number of trainees per institution and per training topic. INSP, national institute of public health; CVDL, central veterinary diagnostic laboratory; CMC, medical communal center; VHFPL, viral hemorrhagic fever project laboratory; IgDHL, ignace deen hospital laboratory; NTBLab, national tuberculosis laboratory; MoF, ministry of fisheries; MoH, ministry of health; BRM & QM, biorisk management and quality management.

A total of 30 participants took part in the biorisk and quality management training. Twelve of the participants were from the National Institute for Public Health (INSP); five were from the Ignace Deen Hospital Laboratory (one of two national hospitals in Conakry); two worked at the National Tuberculosis Laboratory; one headed the MoHH's Directorate of Laboratories, which oversees laboratories and laboratory technician training within the country. Outside of the MoHH, six participants belonged to the Viral Hemorrhagic Fever Project Laboratory (VHFPL), which is under the Ministry of Higher Education and Research, three participants were affiliated with the Ministry of Livestock and Animal Production's Central Veterinary Diagnostic Laboratory (CVDL), and one participant reported to the Ministry of Fisheries (Figure 2).

For molecular diagnostics training, a total of twelve participants were enrolled. Seven of the attendees were affiliated with the National Institute for Public Health (INSP), two participants served at the Communal Medical Center laboratory, and three participants were from the Central Veterinary Diagnostic Laboratory (Figure 2).

### Training Impact and Sustainability

The events targeting leadership personnel focused on establishing and maintaining resilient biorisk and quality management systems, while staff-level training events focused primarily on implementing best practices and performing diagnostics testing in the laboratories. As much as possible, the interrelatedness of biorisk management and quality management was emphasized in order to highlight the advantages of integrating the two systems.

**Figure 3:**
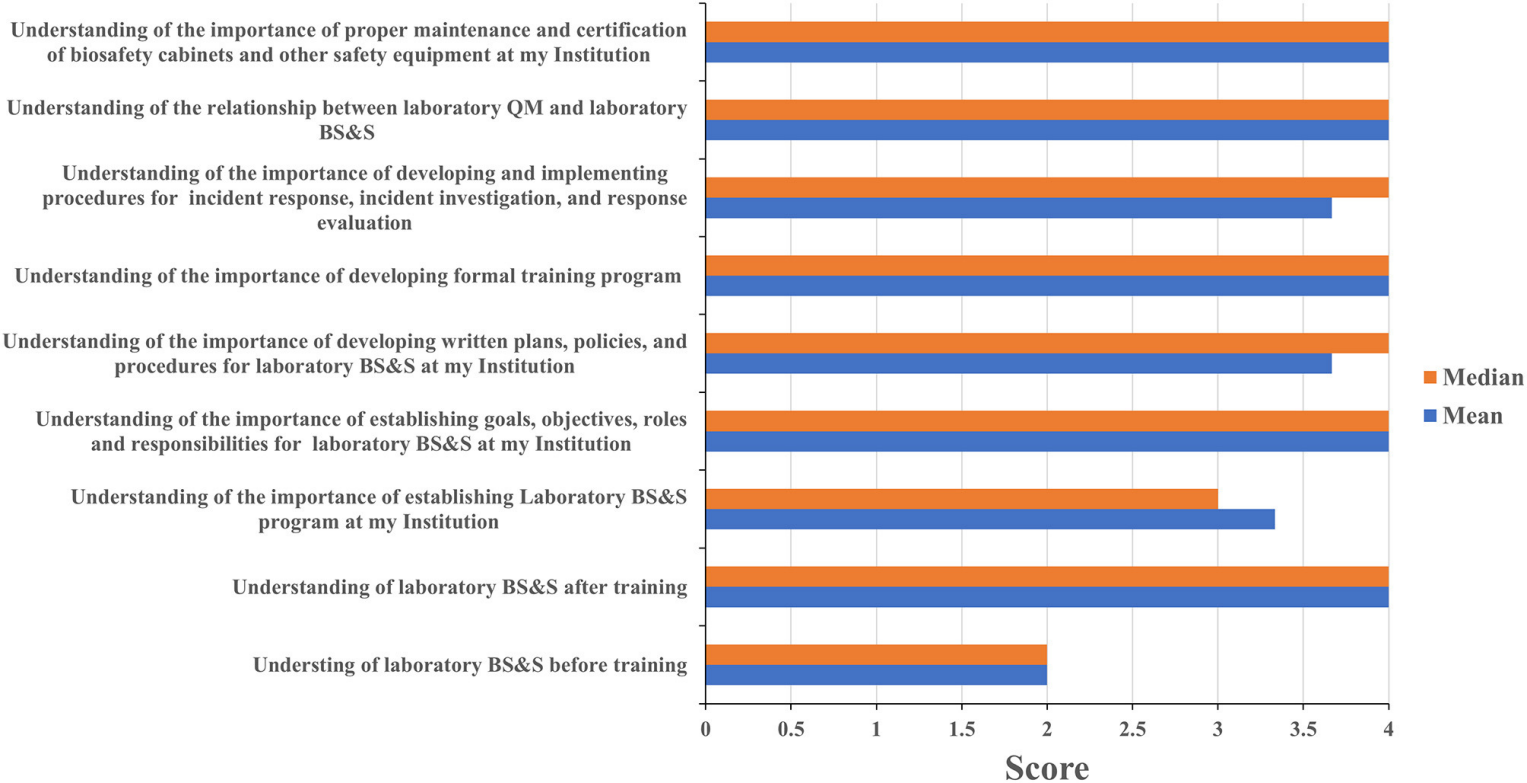
Leadership training impact. Questionnaires were designed for self-evaluation of leadership participant's knowledge impact on their professional activities. Trainees were asked to score 1 to 4 with 1 being the least score and 4 the highest. The mean and median were then calculated. MoH, ministry of health; BS&S, biosafety and biosecurity; QM, quality management.

**Figure 4:**
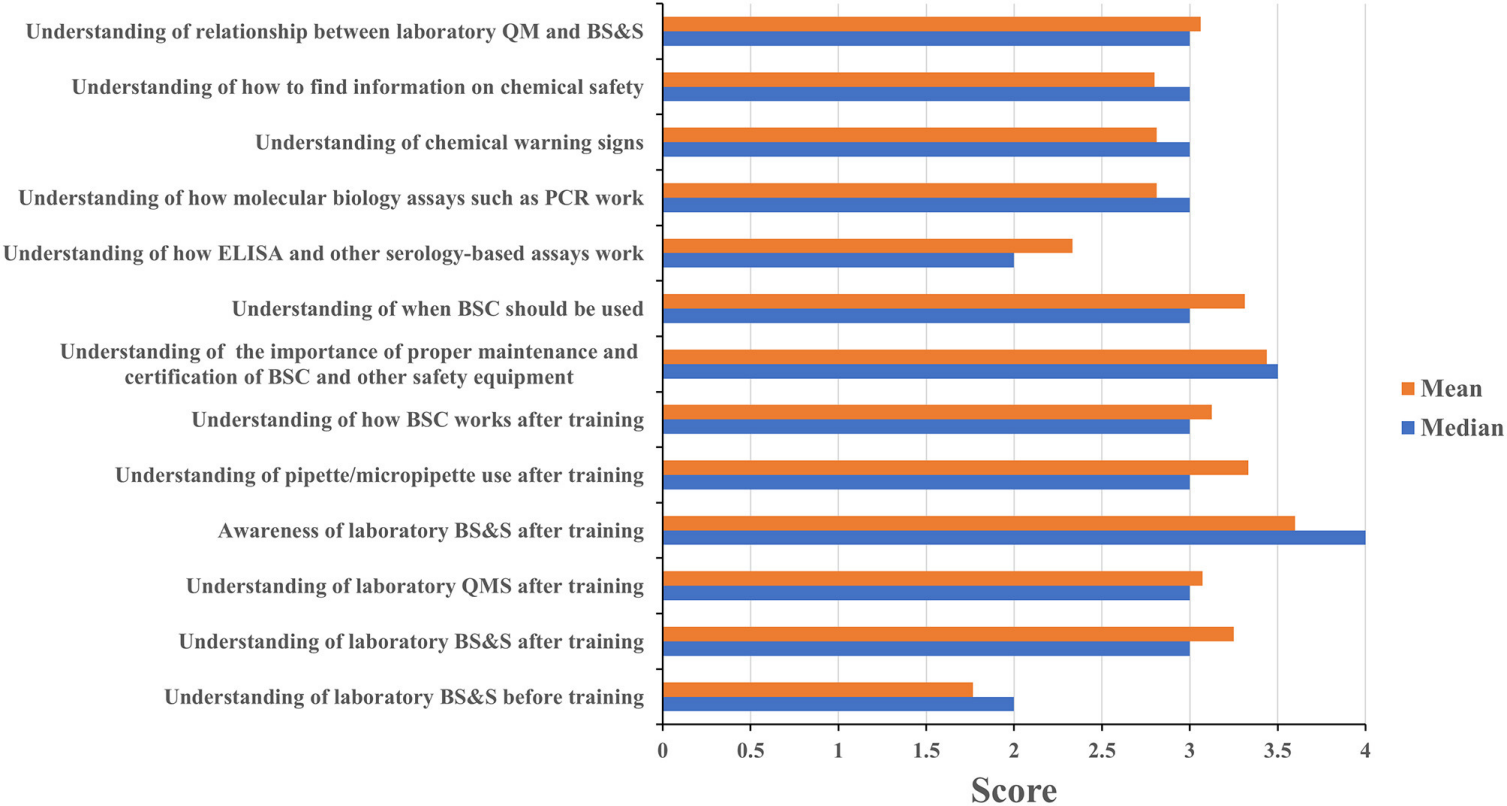
Staff training impact. Questionnaires were designed for self-evaluation of staff participant's knowledge impact on their professional activities. Trainees were asked to score 1 to 4 with 1 being the least score and 4 the highest. The mean and median were then calculated. MoH, ministry of health; BS&S, biosafety and biosecurity; QM, quality management.

Four of eleven leadership event participants and sixteen of twenty staff event participants completed the assessment questionnaire. Leadership and staff participants all reported having a poor understanding of biorisk management prior to attending their respective events with mean/median scores of 2/2 and 1.75/2, respectively; these scores increased to 4/4 among leadership event participants (Figure 3) and 3.25/3 for staff participants (Figure 4) after training attendance. Leadership participants reported increases in understanding for the majority of the key topics in their events (Figure 3). Staff participants reported having very little knowledge of how to perform molecular diagnostics prior to attending the training events. Their understanding of and ability to perform different tasks associated to molecular diagnostics increased to 2.75/3 as a result of the training. The one topic where the majority of staff participants reported the least increase in understanding was for how enzyme-linked immunosorbent (ELISA) assays and other serology-based assays work (mean 2.36, median 2), a topic covered briefly during the staff-level training (Figure 4). Subsequent interactions with laboratorians performing serology-based diagnostics indicated minimum to no prior exposure to the fundamentals of immunology and serology through their education and training by other partners and this could have contributed to the least improvements recorded for the serology-based assays topic.

**Figure 5:**
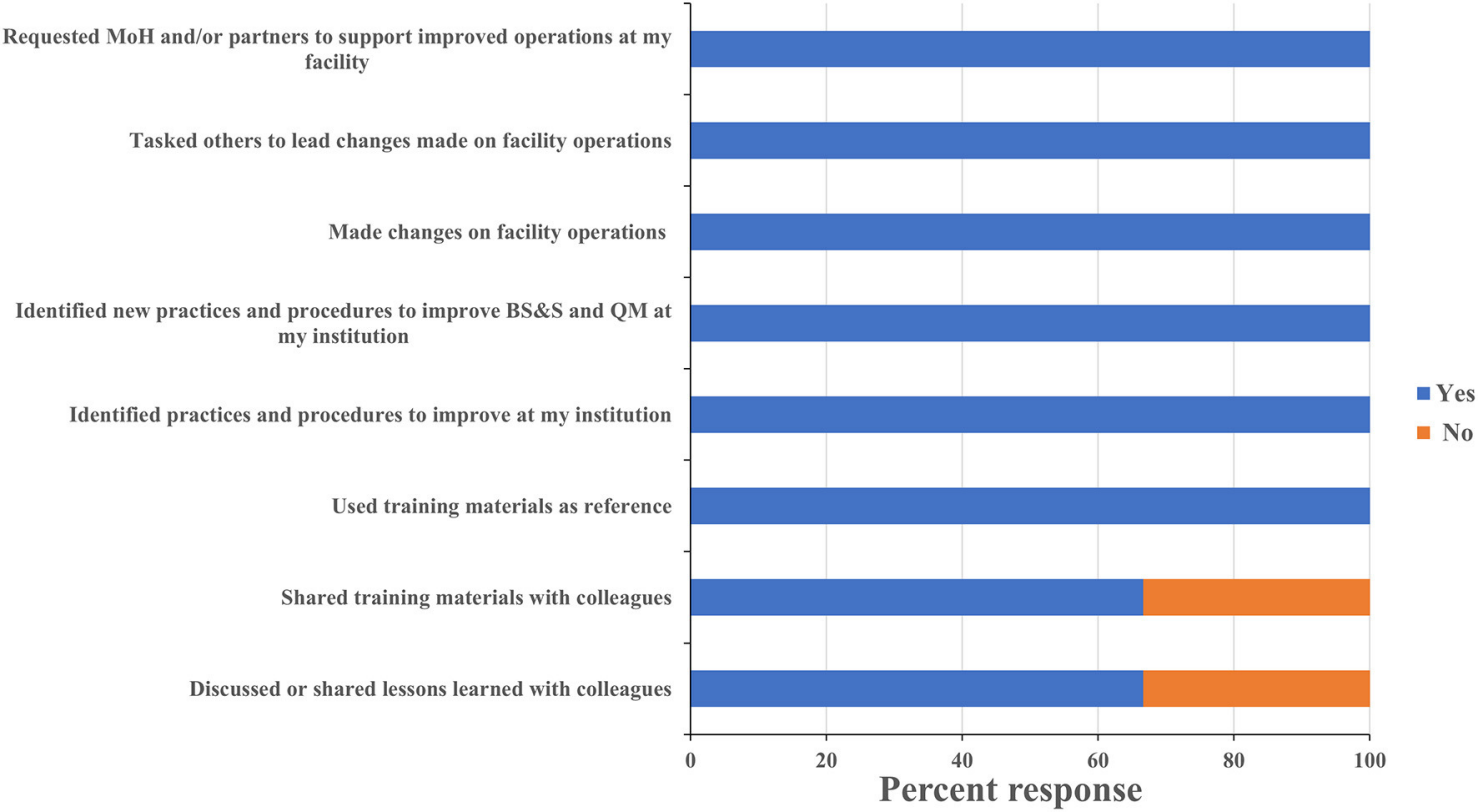
Leadership training sustainability. Questionnaires were designed to assess the sustainability of the lessons learnt and skills gained on leadership participant's professional activities at their institution. Trainees were asked to respond “yes” or “no” to each question and the percentage of respondents selecting each answer was calculated. MoH, ministry of health; BS&S, biosafety and biosecurity; QM, quality management.

**Figure 6:**
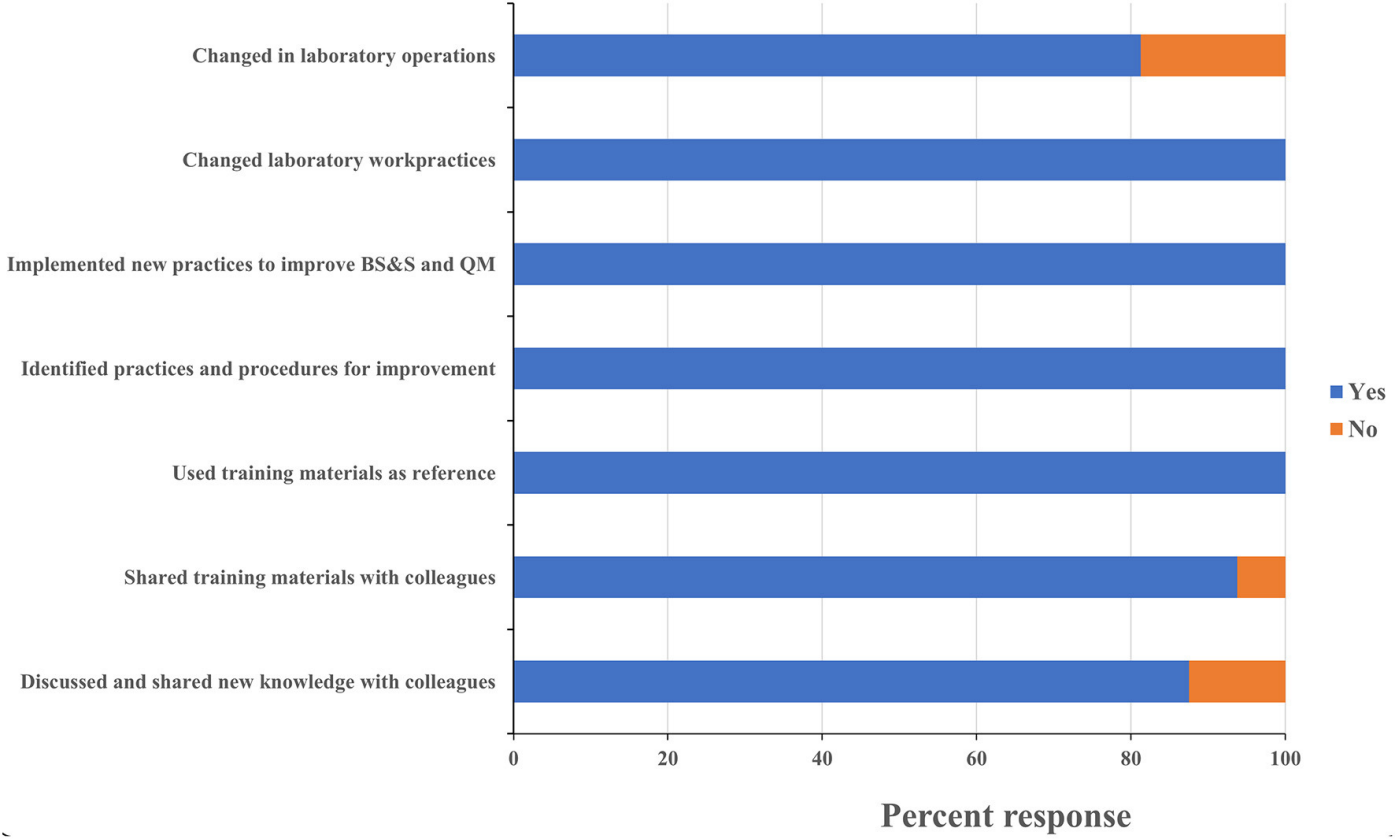
Staff training sustainability. Questionnaires were designed to assess the sustainability of the lessons learnt and skills gained on staff participant's professional activities at their institution. Trainees were asked to respond “yes” or “no” to each of these questions and the percentage of respondents selecting each answer was calculated. MoH, ministry of health; BS&S, biosafety and biosecurity; QM, quality management.

One persistent challenge with workshop-based training on such significant topics as biorisk and quality management is the difficulty in determining what impact the training has on actual behaviors and operations in the workplace, and evaluating whether those impacts are persistent or sustainable. All leadership respondents reported having identified existing practices and procedures that could be improved, and identified new ones that should be implemented based on what they learned during the training. Leadership respondents all reported that the training positively influenced requests for support they made to their ministry and to international partners (Figure 5). One director, for example, requested his fellow participants' inputs on the safety features of a laboratory renovation project that was about to commence at his facility and then took the resulting recommendations back to the partner funding the work. An additional indicator that leadership participants were positively influenced by the training was the designation of Biosafety and Quality Officers at the CVDL and INSP and the increasing adoption of a culture of using SOPs to ensure protocol standardization at the INSP. These scores and anecdotes suggest that the impact the training had on laboratory safety and quality operations extended well-beyond the individuals who directly participated in the training program. All staff respondents reported making changes to how they worked in their home laboratories, implemented new practices to improve biorisk and quality management, and identified new ones that should be implemented as a result of what they learned at the training (Figure 6). As an example of this, one staff-level participant reported that they used what they learned in the course to recommend changes to the location of a new biosafety cabinet that was being installed in their laboratory in order to reduce airflow disruptions to the unit. Eighty eight percent of the staff-level participants reported discussing what they learned at the events with their colleagues, 80% reported implementing changes to how their home laboratories operated (for example use of BSC to process infectious materials, wearing appropriate PPE when working or handling infectious materials, use of SOP to execute a testing procedure etc.…) and 94% shared the printed materials they received with their colleagues, which included copies of the presentations used and sample standard operating procedures (SOPs) (Figure 6). These data suggest that the diagnostics trainings also had an impact on overall laboratory operations that extended beyond the individuals who participated directly.

### Implementation of Laboratory Diagnostic Testing

**Figure 7:**
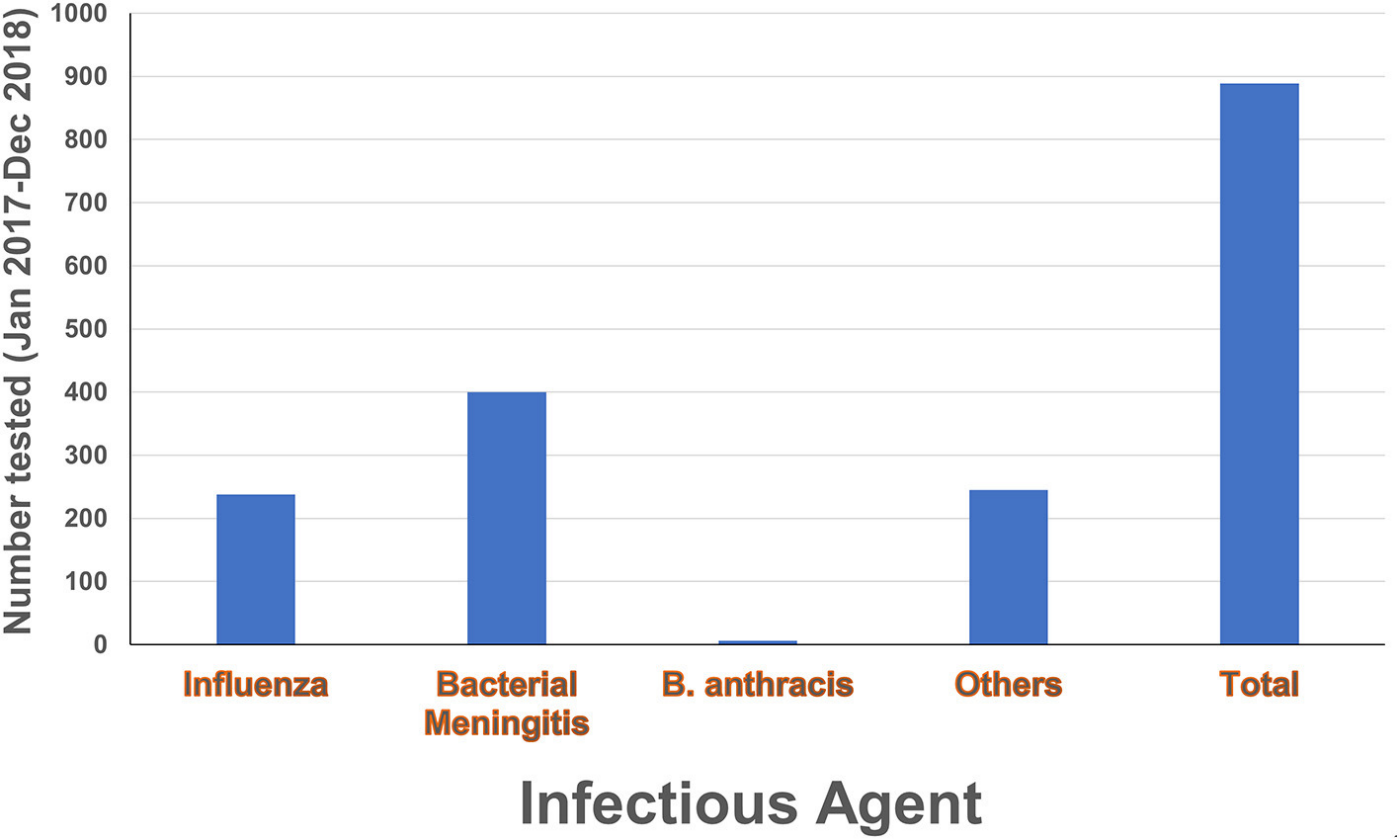
Diagnostic testing activities. Number of tests conducted in the laboratory during 2017–2018 by local laboratory personnel under the mentorship of the country Program Lead. Diagnostic activities included anthrax testing, bacterial meningitis testing and sero-grouping, and influenza testing and subtyping. The category “Others” refers to multiplex PCR testing for yellow fever virus, Dengue virus, Chikungunya virus, West Nile virus, *Coxiella burnetii*, Plasmodium, Rickettsia, *Streptococcus pneumonia*, Brucella, Leptospira and Salmonella.

The Nongo laboratory was intended to serve as the molecular diagnostics annex for INSP. As discussed earlier, the country Program Lead was embedded with the Guinean staff assigned to the laboratory to provide expertise, mentorship, and oversight as the laboratory became operational. Building off the theoretical backgrounds provided during the biorisk and quality management trainings, as well as the formal diagnostics training events, the country Program Lead guided the assigned laboratory personnel through the process of establishing workflows, provided individual coaching on specific laboratory skills, and helped the laboratorians develop their troubleshooting capacity. The laboratory originally focused on EVD testing, with the test menu quickly expanding to include diagnostic testing and surveillance for bacterial meningitis, influenza and other respiratory viruses, and anthrax (Figure 7 and Table 1) thanks to investments by partners such as the WHO and the United States Centers for Disease Control and Prevention (US-CDC). To further reinforce and broaden the molecular detection capacity at the laboratory and to satisfy the demand of the country to develop capacity to diagnose Guinea's identified priority diseases, the Program Lead helped the laboratorians implement a multiplexed molecular assay for the simultaneous detection of agents associated with tropical fever in Africa including *Brucella* species, Yellow fever virus, *Streptococcus pneumoniae, Coxiella burnetii* Dengue virus, Chikungunya virus, West Nile virus, *Plasmodium, Rickettsia, Leptospira*, and *Salmonella* (Figure 7 and Table 1). This newly developed capacity was beneficial for the later implementation of a multiplex panel for the detection of respiratory viruses.

**Table 1:** Diagnostic and surveillance capacity available at the INSP Nongo laboratory at the end of the project.

**Disease**	**Diagnostic and Surveillance capacity**		**SOP available**
	**Before Project**	**After Project**	
Anthrax	No	Yes	Yes
Ebola Virus Disease	No	Yes	Yes
Influenza	No	Yes	Yes
Influenza typing	No	Yes	Yes
Meningitis	No	Yes	Yes
Meningitis grouping	No	Yes	Yes
Dengue fever	No	Yes	Yes
Yellow fever	No	Yes	Yes
Chikungunya	No	Yes	Yes
West Nile	No	Yes	Yes
Q fever	No	Yes	Yes
Brucellosis	No	Yes	Yes
Typhoid fever	No	Yes	Yes
Malaria	No	Yes	Yes
Leptospirosis	No	Yes	Yes

Generally, it was observed that partners donating reagents and consumables to support new testing would provide a minimal amount of training on performing the assays, with little time spent addressing troubleshooting and validation of the assays or integrating them into existing workflows. Moreover, some reagents were provided without any additional training. In such circumstances, the Program Lead worked with the Nongo laboratory leadership and personnel to address these gaps by developing their own SOPs for new tests, validating the assays, and determining how to best integrate new tests into the laboratory's existing workflow (**Table** and **Figure**). Addressing these topics positioned the laboratory to be able to implement new tests in the future without extensive partner training and thereby improving laboratory independence and capability. As of December 2018, roughly nine hundred molecular tests had been performed at the laboratory by local laboratory personnel under continuous mentorship and oversight of the Program Lead (Figure 7 and Table 1).

One often-overlooked component to maintaining the operational status of a laboratory in sub-Saharan Africa is the engineering support for the Facility. The Nongo laboratory, for example, included its own generators; several containerized “rooms” with individual electrical, ventilation, and air conditioning systems; an incinerator; and shower and laundry facilities. A failure in any of these systems would severely compromise, if not abolish. The ability of the laboratory to function as intended. To address this knowledge gap, project engineers provided quarterly mentorship to two Guinean maintenance engineers in the form of on-the-job training. Emphasis was placed on routine and periodic maintenance requirements, site and equipment maintenance, and systems troubleshooting and repair.

## Discussion

Laboratory testing is critical for disease diagnostics, disease surveillance, and outbreak response but has been the weak arm of public health systems in Western Africa (19–21). Factors contributing to this weakness include poor infrastructure, a lack of qualified staff, insufficient equipment maintenance, poor quality management systems, poor biorisk management practices, and inadequate development of and adherence to SOPs and other essential documents (19, 22–24). While multiple international and non-governmental partners have invested significant resources into capacity building efforts in the RoG and elsewhere, we have observed that there is a piecemeal approach to addressing knowledge and system gaps. This often results in the training recipients treating each new lesson or skill as unique, rather than as a building block of a broader diagnostic portfolio and set of laboratory standards. Additionally, the laboratorians may fail to realize potential synergies between their activities, leading to inefficiencies and the perpetuation of safety and quality issues.

Tasked with assisting the stand up and establishment of the RoG's first national molecular diagnostics laboratory, we sought to reduce the risk of compartmentalization by providing an integrated, multidisciplinary approach to biorisk management, quality management, and the performance of diagnostics that emphasized the interrelatedness of the topics. Classroom and laboratory-based training opportunities were provided not only to the Nongo laboratory staff, but to laboratory management and staff from multiple human and animal health laboratories in Conakry. Broadening the audience of the events created a pool of individuals positioned to share the skills and knowledge acquired during the training, driving sustained improvements across multiple national-level facilities in the country. The addition of engineering mentorship helped address concerns about the Nongo laboratory equipment and infrastructure remaining functional after standup was complete.

Of particular importance to the success of our effort was the imbedding of the country Program Lead with the Nongo laboratory personnel to serve as mentor. This strategy allowed the program to more effectively coordinate efforts with other partners in addition to permitting day-to-day mentoring and improvement of the skills of the laboratory personnel. The country Program Lead worked with the Nongo laboratory leadership to align and integrate efforts by other implementing partners such as the US-CDC and the WHO. This limited the compartmentalization of practices at the new facility and allowed these additional training efforts to build on the existing skill sets to support the integration of new diagnostics for surveillance of priority diseases. The end result was a marked increase in diagnostic and surveillance capabilities offered by INSP (25, 26).

The self-evaluation approach used to gauge training impact and sustainability is susceptible to bias introduced by the respondents over-reporting their successes. The continued operation of the INSP Nongo laboratory after the program's completion provides a less-biased indicator of the program's success. For example, prior to starting this project, there was no established influenza surveillance activity in Guinea and during the course of this project and as of the date of this submission, the INSP Nongo laboratory continuously posts influenza surveillance data on a weekly basis to the WHO global influenza surveillance and response system (GISRS), indicating that surveillance and reporting efforts remain intact. However, this reporting was interrupted for several months while the laboratory started supporting the COVID-19 pandemic response before resuming after INSP Nongo laboratory staff helped to establish additional COVID-19 testing sites in the country. The laboratory performed the first molecular confirmation of *Bacillus anthracis* in the country (26), and has led the RoG's testing response to the ongoing COVID-19 pandemic (27). The INSP Nongo laboratory continues to broaden its testing capabilities and pursue research opportunities, indicating that the startup and establishment of operations process was successful.

The COVID-19 experience underscores challenges that could arise when it comes to simultaneous handling of multiple disease outbreaks. This insufficiency was likely due to budget and personnel limitations. Therefore, future interventions should be directed toward additional workforce development and establishing mechanisms for funding acquisitions.

## Data Availability Statement

The original contributions presented in the study are included in the article/Supplementary Material, further inquiries can be directed to the corresponding authors.

## Ethics Statement

Ethical approval for this study and written informed consent from the participants of the study were not required in accordance with local legislation and national guidelines.

## Author Contributions

JN, SS, BK, CA, JC, JA, MM, LP, SP, JM, NW, CR, and SA contributed to the development, implementation of the project, and reviewed and approved the final manuscript. JN drafted the manuscript and SA reviewed the initial draft of the manuscript. All authors contributed to the article and approved the submitted version.

## Conflict of Interest

The authors declare that the research was conducted in the absence of any commercial or financial relationships that could be construed as a potential conflict of interest.

## References

[1] Fauci AS Morens DM. The perpetual challenge of infectious diseases. N Engl J Med. (2012) 366:454–61. 10.1056/NEJMra1108296 22296079

[2] Morens DM Fauci AS. Emerging infectious diseases: threats to human health and global stability. PLoS Pathog. (2013) 9:e1003467. 10.1371/journal.ppat.1003467 23853589 PMC3701702

[3] Piot P Soka MJ Spencer J. Emergent threats: lessons learnt from Ebola. Int Health. (2019) 11:334–7. 10.1093/inthealth/ihz062 31385587

[4] World Health Organization. One Year Into the Ebola Epidemic: A Deadly, Tenacious and Unforgiving Virus. Available online at: https://www.who.int/csr/disease/ebola/one-year-report/introduction/en/ (accessed September 20, 2020).

[5] Chan EH Brewer TF Madoff LC Pollack MP Sonricker AL Keller M. Global capacity for emerging infectious disease detection. Proc Natl Acad Sci USA. (2010) 107:21701–6. 10.1073/pnas.1006219107 21115835 PMC3003006

[6] Moon S Leigh J Woskie L Checchi F Dzau V Fallah M. Post-Ebola reforms: ample analysis, inadequate action. BMJ. (2017) 356:j280. 10.1136/bmj.j280 28115316

[7] Moon S Sridhar D Pate MA Jha AK Clinton C Delaunay S. Will Ebola change the game? Ten essential reforms before the next pandemic. The report of the Harvard-LSHTM Independent Panel on the Global Response to Ebola. Lancet. (2015) 386:2204–21. 10.1016/S0140-6736(15)00946-0 26615326 PMC7137174

[8] Centers for Disease Control and Prevention. 2014-2016 Ebola Outbreak in West Africa. Available online at: https://www.cdc.gov/vhf/ebola/history/2014-2016-outbreak/index.html (accessed September 20, 2020).

[9] World Health Organization. Ebola Situation Report. Geneva: World Health Organization (2016).

[10] The World Bank. 2014-2015 West Africa Ebola Crisis: Impact Update. Available online at: http://www.worldbank.org/en/topic/macroeconomics/publication/2014-2015-west-africa-ebola-crisis-impact-update (accessed September 20, 2020).

[11] World Health Organization. International Health Regulations 2005, third edition, Geneva. Available online at: https://www.who.int/ihr/publications/9789241580496/en/ (accessed September 20, 2020).

[12] International Health Regulations 2005. IHR Core Capacity Monitoring Framework: Checklist and Indicators For Monitoring Progress in the Development of IHR Core Capacities in State Parties. Available online at: https://apps.who.int/iris/bitstream/handle/10665/84933/WHO_HSE_GCR_2013.2_eng.pdf;jsessionid=D014FA9D92B79516804D693B5A5931F4?sequence=1 (accessed September 20, 2020).

[13] World Health Organization. Implementation of the International Health Regulations. (2005). Available online at: http://apps.who.int/gb/ebwha/pdf_files/EB136/B136_22-en.pdf (accessed September 20, 2020).

[14] United Nations General Assembly. Sixty-ninth Session Agenda Item 124 Global Health and Foreign Policy, United Nations, Geneva Switserland. Available online at: https://www.who.int/workforcealliance/media/news/2015/UNres2015-health-foreign-policy-group.pdf?ua=1 (accessed September 20, 2020).

[15] World Health Organization. Laboratory Quality Management System Training Toolkit. Available online at: https://www.who.int/ihr/training/laboratory_quality/doc/en (accessed October 14, 2020).

[16] European Committee for Standardization. CEN Workshop Laboratory Biorisk Management Standard 15793 (CWA15793). Brussels: European Committee for Standardization (2011).

[17] World Health Organization. Laboratory Biosafety Manual. 3th ed. St. Geneva: World Health Organization (2004).

[18] International Standard ISO 15189. Medical Laboratories - Requirements for Quality and Competence. 3th ed. St. Geneva: International Organization for Standardization (2012).

[19] Nkengasong JN Nsubuga P Nwanyanwu O Gershy-Damet GM Roscigno G Bulterys M. Laboratory systems and services are critical in global health: time to end the neglect? Am J Clin Pathol. (2010) 134:368–73. 10.1309/AJCPMPSINQ9BRMU6 20716791 PMC7109802

[20] Nkengasong JN Yao K Onyebujoh P. Laboratory medicine in low-income and middle-income countries: progress and challenges. Lancet. (2018) 391:1873–5. 10.1016/S0140-6736(18)30308-8 29550031 PMC5962422

[21] Kelly-Cirino CD Nkengasong J Kettler H Tongio I Gay-Andrieu F Escadafal C. Importance of diagnostics in epidemic and pandemic preparedness. BMJ Glob Health. (2019) 4:e001179. 10.1136/bmjgh-2018-001179 30815287 PMC6362765

[22] Petti CA Polage CR Quinn TC Ronald AR Sande MA. Laboratory medicine in Africa: a barrier to effective health care. Clin Infect Dis. (2006) 42:377–82. 10.1086/499363 16392084

[23] Nkengasong JN Mesele T Orloff S Kebede Y Fonjungo PN Timperi R. Critical role of developing national strategic plans as a guide to strengthen laboratory health systems in resource-poor settings. Am J Clin Pathol. (2009) 131:852–7. 10.1309/AJCPC51BLOBBPAKC 19461093

[24] Kebede S Gatabazi JB Rugimbanya P Mukankwiro T Perry HN Alemu W. Strengthening systems for communicable disease surveillance: creating a laboratory network in Rwanda. Health Res Policy Syst. (2011) 9:27. 10.1186/1478-4505-9-27 21702948 PMC3142247

[25] Organization WH. Global Influenza Surveillance and Response System. Available online at: https://www.who.int/influenza/gisrs_laboratory/flunet/en/ (accessed September 20, 2020).

[26] Keita MB Ndjomou J Tohonamou P Traore B Mamady S Keita M. The first laboratory-confirmed case of anthrax in Guinea. Glob Bio. (2020) 1. 10.31646/gbio.74

[27] Organization WH. WHO World Emergency Dashboard WHO (COVID-19). Available online at: https://covid19.who.int/region/afro/country/gn. (accessed July 18, 2020).

