# Supplementary material for: Sustainable Laboratory Capacity Building After the 2014 Ebola Outbreak in the Republic of Guinea
Source: Front Public Health. 2021 Jun 4;9:659504. doi: 10.3389/fpubh.2021.659504 (PMC8220810; doi:10.3389/fpubh.2021.659504)
Supplement: Supplementary file 1 [file Table_1.DOCX]

| **Question** | **Score (1-4)*** | | | |
| --- | --- | --- | --- | --- |
|  | **1** | **2** | **3** | **4** |
| Before the training, I understood laboratory biosafety and laboratory biosecurity very well. |  |  |  |  |
| I now have a better understanding of laboratory biosafety and laboratory biosecurity. |  |  |  |  |
| I am more aware of laboratory biosafety and laboratory biosecurity since I took the training. |  |  |  |  |
| I now have a better understanding of the importance of establishing a program to manage laboratory biosafety and laboratory biosecurity at my facility. |  |  |  |  |
| I now have a better understanding of the importance of establishing clear goals, objectives, roles, and responsibilities for laboratory biosafety and laboratory biosecurity at my facility. |  |  |  |  |
| I now have a better understanding of the importance of developing written plans, policies, and procedures for laboratory biosafety and laboratory biosecurity at my facility. |  |  |  |  |
| I now have a better understanding of the importance of developing a formal training program that tracks all of the training my personnel receive. |  |  |  |  |
| I now have a better understanding of importance of developing and implementing procedures for incident response, incident investigation, and response evaluation. |  |  |  |  |
| I now have a better understanding of the relationship between laboratory quality management and the management of laboratory biosafety and laboratory biosecurity. |  |  |  |  |
| I now have a better understanding of why proper maintenance and certification of biosafety cabinets and other safety equipment is important. |  |  |  |  |

*Score*: 1 = strongly disagree and 4 = Strongly agree. Leave blank for no opinion or N/A (not available)*

| **Question** | **Score (1 or 0)** | |
| --- | --- | --- |
|  | **1** | **0** |
| I have discussed or shared what I learned at the training with my colleagues |  |  |
| I have shown or shared the printed materials I received at the training with my colleagues |  |  |
| Since the training, I have used the materials I received as a reference to help me answer a question. |  |  |
| Since the training, I have used the materials I received as a reference to help me remember important information. |  |  |
| Did the training help you identify practices and procedures at your facility that you think could be improved? |  |  |
| Did the training help you identify new practices and procedures that you think should be implemented at your facility to improve laboratory biosafety, laboratory biosecurity, and/or laboratory quality management? |  |  |
| Have you begun making changes to how your facility operates as a result of what you learned at the training? |  |  |
| Have you tasked others with making changes to how your facility operates as a result of what you learned at the training? |  |  |
| Have you made requests to your Ministry and/or other local and international partners for support (financial, equipment, or other) in improving operations at your facility as a result of what you learned at the training? |  |  |

**Score(1,0): 1 for yes and 0 for no*

| **Question** | **Score (1-4)*** | | | |
| --- | --- | --- | --- | --- |
|  | **1** | **2** | **3** | **4** |
| Before training, I understood laboratory biosafety and laboratory biosecurity very well. |  |  |  |  |
| I now have a better understanding of laboratory biosafety and laboratory biosecurity. |  |  |  |  |
| I now have a better understanding of laboratory quality management systems. |  |  |  |  |
| I am more aware of laboratory biosafety and laboratory biosecurity since I took the training. |  |  |  |  |
| I now have a better understanding of how to use an adjustable micropipette/ pipette correctly. |  |  |  |  |
| I now have a better understanding of how a biological safety cabinet (biosafety cabinet) works. |  |  |  |  |
| I now have a better understanding of why proper maintenance and certification of biosafety cabinets and other safety equipment is important. |  |  |  |  |
| I now have a better understanding of how enzyme-linked immunosorbent assays (ELISAs) and other serology-based assays work. |  |  |  |  |
| I now have a better understanding of how molecular biology assays such as polymerase chain reaction work. |  |  |  |  |
| I now have a better understanding of chemical warning labels. |  |  |  |  |
| I now have a better understanding of how to find information about how to use chemicals safely. |  |  |  |  |
| I now have a better understanding of the relationship between quality management, laboratory biosafety and laboratory biosecurity. |  |  |  |  |

*Score*: 1 = strongly disagree and 4 = Strongly agree. Leave blank for no opinion or N/A (not available)*

| **Question** | **Score (1,0)*** | |
| --- | --- | --- |
|  | **1** | **0** |
| I have discussed or shared what I learned at the training with my colleagues. |  |  |
| I have shown or shared the printed materials I received at the training with my colleagues. |  |  |
| Since the training, I have used the materials I received as a reference to help me answer a question. |  |  |
| Since the training, I have used the materials I received as a reference to help me remember important information. |  |  |
| Did the training help you identify practices and procedures at your institution that you think could be improved? |  |  |
| Did the training help you identify new practices and procedures that you think should be implemented at your institution to improve laboratory biosafety, laboratory biosecurity, and/or laboratory quality management? |  |  |
| Have you made changes to how you personally work in the laboratory as a result of what you learned at the training? |  |  |
| Have you made changes to how your laboratory operates as a result of what you learned at the training? |  |  |

**Score(1,0): 1 for yes and 0 for no*
